# Trends and Spatial Patterns of Suicide Among Adolescent in Ecuador, 1997-2016

**DOI:** 10.2174/1745017901814010283

**Published:** 2018-11-08

**Authors:** Solange Núñez-González, A. Gabriela Lara-Vinueza, Christopher Gault, J. Andrés Delgado-Ron

**Affiliations:** Centro de Investigación en Salud Pública y Epidemiología Clínica (CISPEC), Universidad Tecnológica Equinoccial, Quito, 170129, Ecuador

**Keywords:** Suicide, Adolescent, Mortality, Trends, Spatial Regression, Ecuador (source: MeSH NLM), Spatial

## Abstract

**Background:**

Suicide is a global public health problem, ranking among the top 20 leading causes of mortality.

**Objective:**

The aim of the present study is two-fold. Firstly, it describes the temporal trends of suicide in adolescents from 1997 to 2016 in Ecuador, allowing us to identify critical periods. Secondly, it analyzes the spatiotemporal clusters of high mortality rates and the spatial distribution due to suicide in the country, from 2011 to 2016.

**Methods:**

This is an ecological study; we included all death certificates of suicide among adolescents in the 10 - 19 age groups both sex, from the National Institute of Statistics and Census (INEC) database in Ecuador from 1997 to 2016. In order to assess the trend of suicide rates, we obtained Annual Percentage Changes (APCs) and average Annual Percent Changes (AAPCs) through Joinpoint regression analysis. Space-time scan statistics were used to identify high-risk clusters, and the spatial autocorrelation was evaluated through global Moran index.

**Results:**

Suicides at a national level increased from 165 deaths in 1997 to 286 deaths in 2016; rates increased from 12.7 to 23.3 per 100,000 population along with a significant increase of the trend at the national level (AAPC=3.7%; 95% CI: 2.1 to 5.2). We identified two significant spatial clusters for a high occurrence of suicide: the primary most likely cluster included 83 cantons (Risk Relative=2.28) while the second most likely cluster included 20 cantons (Risk Relative=1.74). The Global Moran I index for the study period showed a positive spatial autocorrelation (0.27; p = 0.001).

**Conclusion:**

Suicide rates in adolescents significantly increased over the 20-year study period; the spatial analysis indicates the presence of high occurrence clusters in the Amazon and Southern Highlands regions of the country. This growing phenomenon may be a reflection of the lack of policies and strategies focused on the adolescent’s mental health at a national level, added to factors such as family dysfunction, school failure, vulnerable ethnic groups, and immigration patterns.

## 1 INTRODUCTION

Suicide is a global public health problem, ranking among the top 20 leading causes of mortality. According to the World Health Organization (WHO), every 40 seconds a life is lost due to self-harm; that accounts for 800,000 deaths per year, 1.4% of total deaths [1].

People younger than 19 are especially susceptible; suicide was the second leading cause of mortality among females and the third among males in the last decade - 6% of all deaths in 2015 (32,499 girls and 34,650 boys) — were caused by suicide, the majority of them via self-harm [2, 3]. As national reports can't account for all suicides (due to underreporting or misreporting); suicide could be the leading of death worldwide among young people [1].

Suicidal behavior - suicide ideation, planning suicide, attempting suicide, and suicide itself — has the highest rates in low and middle-income countries (LMIC); they account for 75% of youth suicides globally [2]. In America, for instance, 65,000 suicides have been noted per year [4]. A comparison of the last two decades (1990-1999 and 2000–2009) showed the average suicide rate per 100,000 young people in South America increased from 1.04 to 2.32 for boys, and from 1.45 to 2.30 for girls [5]. Guyana, Suriname, and Ecuador have reported the highest rates of suicide in America for girls (Suriname reported the highest rate of suicide for boys only) [5, 6]. Among Andean countries, Ecuador has the second highest suicide rate (7.1 per 100,000 population) after Bolivia (18.7/100,000), and ahead of Colombia (6.1/100,000) and Peru (5.8/100,000).

During the last 15 years, 13,024 suicides were officially reported in Ecuador [7]. Data from the National Institute of Statistics and Census (INEC) indicate that between 1997 and 2016, an average of 191 adolescents died per year [8]. Given the high social stigma associated with suicide, it is likely that current data underestimate actual rates, especially in people younger than 19 years old [4]. Previous studies have looked at the epidemiology of suicide in the Ecuadorian general population [7, 9], but none of them explored the specific trends among teenagers.

The objective of this study is to describe the temporal trend of suicide in adolescents between 10-19 years old, from 1997 to 2016, and to analyze the presence of spatiotemporal clusters of high mortality rates and the spatial distribution due to suicide in adolescents in the country, from 2011 to 2016

## 2 MATERIAL AND METHODS

### 2.1 Data Sources

Data were collected from the National Institute of Statistics and Census (INEC) database, in its “Statistical Reports” section of live birth, death, and fetal death [10].

Death registries include “basic cause” of death coded according to the International Classification of Diseases (ICD), which we used for our temporal analysis. Registries matched ICD-10 codes X60-X84, for suicide. Finally, we extracted population data from estimates of 1997 and 2010 censuses conducted by INEC [11].

### 2.2 Study Area

Ecuador is one of the smaller countries in South America, located on the west side of the Pacific Ocean; total area is 283,561 km^2^ [12]. It is geographically divided into four regions (the Amazon, the Highlands, the Coast, and the Galápagos Islands) and politically split into 24 provinces, which, in turn, are split into 224 cantons [13].

### 2.3 Statistical Analysis

Descriptive statistics were used to describe the data, frequencies, and percentages for categorical variables and mean with standard deviations for continuous variables. The association between monthly distributions of suicide and sex was verified using either Pearson's Chi squared test of independence or the Fisher Exact test.

Suicide rates by sex, age group (10-14 and 15-19 years old), and residency area were calculated. All rates are expressed as death per 100,000 population. Microsoft Excel 2010 (Microsoft Office Professional Plus 2010) was used to calculate suicide rates and standard errors.

### 2.4 Time Trends

We used a Joinpoint regression model to identify the years when there were significant changes in suicide rates. Joinpoint regression analysis fits a series of joined straight lines on a logarithmic scale; straight line segments are joined at “joinpoints”, where mortality trend changes with statistical significance [14]. The slope of each line segment, of the best-fitting model, was expressed as the annual percentage rate change (APC) and average annual percent change (AAPC). Significance tests were performed using the Monte Carlo permutation technique. We considered a p-value statistically-significant when below a confidence level of 0.05. For the statistical analysis, our team used the Joinpoint Regression Program version 4.4.0.0, from the Surveillance Research Program of the US National Cancer Institute [14].

### 2.5 Spatial and Spatial-temporal Cluster Analysis

We identified high-risk spatiotemporal clusters of suicide in Ecuador during the period 2011-2016 using retrospective Kulldorff’s space-temporal scan statistics. Under the statistical assumption that mortality cases follow a Poisson distribution, we used a discrete model. The 224 cantons of Ecuador were our spatial unit of analysis, with a maximum spatial cluster size of 30% of the population at risk, and a maximum temporal cluster size of 50% of the study period. Primary and secondary clusters were detected through the log likelihood ratio (LLR) test [15]. The statistical significance of these clusters was calculated through Monte Carlo simulations. We analyzed spatiotemporal trends using SaTScan software [16] and displayed them in cartographic representations created by free software QGIS 2.18.14.

### 2.6 Spatial Distribution

Finally, using Global Moran’s I index, we assessed the presence of global spatial autocorrelation. We calculated the average annual crude mortality rates by cantons for the period 2011-2016, to both correct for random fluctuations and to provide greater stability of mortality rates in small cantons. We calculated smoothed mortality rates by applying the Local Empirical Bayesian smoothing method [17]. We used Local Index of Spatial Association (LISA) by means of Local Moran’s I index to evaluate the existence of local autocorrelation. Thus, identifying significant hot spots (high values next to high, HH), cold spots (low values next to low, LL), and spatial outliers (high amongst low, HL or vice versa, LH) of mortality rates [18]. For spatial representation of the Local Moran’s index, we created Moran Maps that include cantons with significant differences. GeoDa software was used for the spatial analysis and smoothed mortality rates calculation (GeoDa Center for Geospatial Analysis and Computation, Arizona State University, Tempe, AZ, USA). Additional cartographic representations were created to showcase this analysis.

## 3 RESULTS

3,824 people between 10-19 years committed suicide in Ecuador during the investigated period (1997–2016), out of which 52.3% of deaths occurred in males (n=1,999) and 47.7% in females (n=1,825). There were 4 registered suicide cases for children under 10 years of age in 2015, two suicides in children 8 years old and two suicides in children 9 years old. Stratified by area of residence, 67.1% (n=2,565) lived in urban areas, 29.9% (n=1,144) in rural areas, and 3% (n=115) in unspecified areas. Intentional self-harm by hanging, strangulation, and suffocation represented 52% of all cases (n=1,986). Self-poisoning and exposure to other and unspecified chemicals and noxious substances represented 21.9% of cases (n=840), while self-poisoning and exposure to pesticides represented 17.3% of cases (n = 662). These three ICD-10 codes (X70, X69, and X68, respectively) accounted for 91.2% of suicides (Table **1**).

We did not find significant differences between the monthly distribution of suicides and sex (p=0.14).

### 3.1 Time Trends

Suicides, in absolute numbers, increased at the national level from 165 deaths in 1997 to 286 in 2016. Between these years, suicide rates increased from 12.7 to 23.3 per 100,000 population, significantly increasing the trend at the national level (AAPC: 3.7%; 95% CI: 2.1 to 5.2).

Among males, rates significantly increased from 5.07 to 8.79 per 100,000 population over the 20-year study period (AAPC: 2.5%; 95% CI: 0.6 to 4.3). Rates stratified by the area of residence showed APC increased 3.05% per year (1997-2016; p=0.004) in urban areas and 5.32% per year (1997-2016; p<0.001) in rural areas. Female rates reported a non-significant increase from 7.04 to 9.19 per 100,000 population (AAPC: 1.7%; 95% CI: -0.1 to 3.5). APC for urban areas was 0.92% per year (1997-2016; p=0.32) and 1.79% per year (1997-2016; p=0.14) in rural areas. The joinpoint analysis according to sex, age group, and area of ​​residence is described in Table **2**.

### 3.2 Spatial-temporal Cluster Analysis

**Fig. (1):**
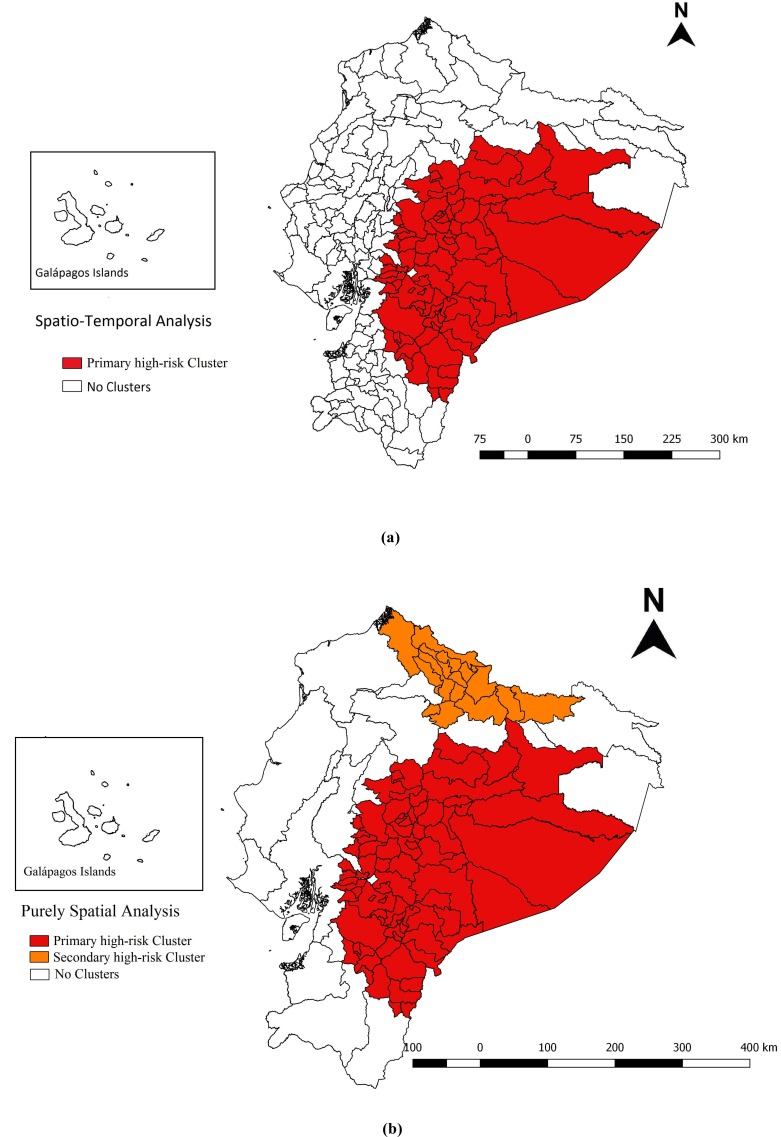
Suicide rates by cantons in Ecuador, 2011 to 2016. **a**) Spatio-temporal cluster analysis. **b**) Purely spatial cluster analysis.

Our space-temporal analysis identified one significant cluster for high occurrence of suicide in the years from 2011 to 2013; it included 83 cantons in 13 provinces (Bolivar, Cotopaxi, Chimborazo, Napo, Pastaza, Tungurahua, Orellana, Azuay, Cañar, El Oro, Guayas, Morona Santiago, and Zamora Chinchipe), totaling 295 suicides; with a RR of 2.51 (LLR: 81.07; p <0.001), and an annual mortality rate of 3.1 per 100,000 population (Fig. **1a**).

### 3.3 Spatial Cluster Analysis

We identified two significant spatial clusters for a high occurrence of suicide, between 2011 and 2016. The primary high-risk cluster included the same cantons and provinces reported in our previous space-temporal analysis, totaling 576 deaths; RR was 2.89 (LLR: 168.72; p<0.001) and the annual mortality rate was 2.9 per 100,000 population. The secondary high-risk cluster included 20 cantons in 5 provinces (Carchi, Esmeraldas, Imbabura, Pichincha, and Sucumbíos), totaling 131 suicides; RR was 1.74 (LLR: 15.57; p<0.001) and the annual mortality rate was 2.3 per 100,000 population (Fig. **1b**).

### 3.4 Spatial Distribution

**Fig. (2):**
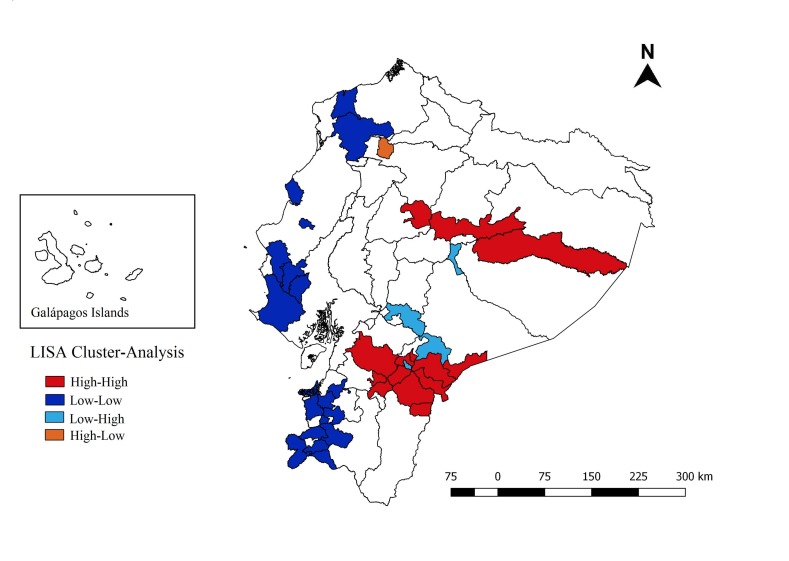
Local Index of Spatial Association (LISA) cluster analysis of suicide rates by cantons in Ecuador, 2011 to 2016.

At the canton level, average annual suicide rates per 100,000 population ranged from 0 to 12.93; smoothed mortality rates ranged from <0.001 to 12.93. The Global Moran I index for the study period shows a positive spatial autocorrelation (0.27; p=0.001). We identified a high-risk cluster (High/High) that included 14 cantons located in 6 provinces (Cotopaxi, Napo, Pastaza, Azuay, Morona Santiago, and Zamora Chinchipe). Clusters with low rates (Low/Low) included 18 cantons located in 6 provinces (Esmeraldas, El Oro, Guayas, Loja, Manabí, and Santa Elena) (Fig. **2**).

## 4 DISCUSSION

We found that suicide among young people increased in Ecuador during the study period; the regression analysis showed a significant growing trend in men living in rural and urban areas, suicide among young women also showed a growing trend although not significant.

Hanging, chemical poisoning, and the ingestion of pesticides caused 90% of all suicides.

Both the spatial-temporal and the spatial analyses identified the same primary high-risk cluster; with most of the provinces located within the cluster in the Amazon region and in the Southern Highlands (RR=2.89); the cluster included the coastal province Guayas. The secondary cluster, located in the north, spans the three main geographical regions of the country. Cotopaxi, Napo, Pastaza, Azuay, Morona Santiago, and Zamora Chinchipe were identified within a high-risk cluster by the Global Moran I index; it should be noted that these six provinces appeared as a high-risk area in three different analyses. Low rates were found mostly in the coastal region.

Our temporal trend findings differ from studies conducted in high-income countries where suicide rates in adolescents have either stabilized or decreased [19]. It is, however, comparable to those of other countries in Latin America [20, 21], as well as those of previous studies in the country. Suicide is highly prevalent in Ecuador, being most common in urban areas [7, 9].

While male suicide rates significantly increased across almost all groups, suicide continues to be the leading cause of death among female adolescents. Female suicide rates increased in the youngest (10-14 years old) group, with most of the effect focused in rural areas. This trend is similar to that found in a study in OECD (Organization for Economic Co-operation and Development) countries where female suicide rates in highly rural countries like China and India exceeded those of males [22]. Male suicides rates also increased significantly in rural areas (10-14 years old APC=5.6% & 15-19 years old APC=4.83%). It is believed that economic, political, and financial problems in the continents during this period contributed to this outcome [6]. Further studies are required to understand the influence of aspects like socio-economic status and gender in these settings.

Suicide in adolescents varies significantly between genders; societal expectations of gender may play a role by emphasizing man’s obligation to be strong, independent, and prone to risks - men, for instance, are less likely to seek help during periods of depression or suicidal behavior [19, 20, 23]. Thus, explaining why men in Ecuador and in other countries kill themselves more often than women. It is unknown whether suicide attempts are more common among women in Ecuador, as suggested by the literature. While a school-based survey by the Ministry of Public Health cited by Svanemyr *et al*. [24] showed that 17.9% of teenagers between 13 and 15 years considered attempting suicide, the study was not segmented by gender. Further studies are required to understand the prevalence of both suicide ideation and suicide attempt.

Hanging and poisoning remain the most common mechanisms of death [7, 9], with males using more violent means than females, and as such being more likely to be successful [25]. Intake of chemicals and harmful substances could be aggravated by the ease of access, low cost, and lack of regulation (especially using organophosphates and carbamates, which are frequently used pesticides in banana and flower plantations) [26, 27].

As shown in our spatial analyses, two regions were at a higher risk: the Amazon region and the Southern Highlands region. Provinces in the Amazon are populated mainly by indigenous people. Suicide rates of indigenous people are considerably higher than those of non-indigenous [28, 29]; this is also true for children and teenagers [19, 30]. Further studies are required to understand the relationship between indigenous vs. non-indigenous risk factors. The Southern Highlands, on the other hand, had the highest migratory density per canton according to the 2001 Population and Housing Census [31]; a previous study looking at the association between parental migration and health behavior found that adolescents (13-18 years) were at higher risk of suicide ideation [32].

A previous study by Ortiz-Prado *et al*. [7] suggested a relationship between high altitudes areas and suicide. However, our results contradict this hypothesis. Most provinces (4 of 6) at high risk in all of our spatial analysis are located in the Amazon, with altitudes between 0 and 200 meters. These authors also suggested family dysfunction, school failure, sexual abuse, alcohol, drug use, and immigration patterns could aggravate suicides. While this escapes the scope of our analysis, we can assert that we did not observe significant variations between months, which might have been related to school failure — students are exposed to stress factors including a new environment, new academic expectations, financial hardships, and new schedules [33]. Researchers should look at these and other phenomena, such as malnutrition [34], child abuse [35] or high migratory flows to gain insights on the causes of suicide in the country.

Only 1.2% of the budget of the Ministry of Public Health (MSP) was directed towards mental health strategies; 59% of this amount was allocated to psychiatric hospitals distributed across the country [36]. In 2014, 270 psychiatrists worked in public health facilities - 45 less than in 2002 - and were distributed as follows: 162 in the Highlands, 103 in the Coast, and 5 in the Amazon; there were no specialists in the Galápagos Islands [37] - a significant shortage of access to mental health services. The budget deficit, lack of national preventive strategies, and a limited number of ​​mental health professionals might have worsened suicide in the country. As of 2016, the Ministry proposed to establish an intervention strategy for people with suicidal ideation and self-inflicted injuries in coordination with educational institutions, community, and family members. Such a strategy includes preventive tasks, comprehensive health care, as well as guidance and management to family members [38]. In spite of these actions, it is unlikely that such strategies will achieve a ten percent reduction in suicide rates by 2020, as proposed by WHO's comprehensive mental health action plan.

Our study is strengthened by the use of jointpoint regression model; which avoids the pre-specification of periods by the researcher. The statistical method developed by Kulldorf identifies the distribution of the disease in time and space, evaluating the significance of clusters in high-risk, low-risk, and all-risk categories; as well as an early detection of epidemics in epidemiological surveillance [39].

Our study was limited by the difficulty in establishing an association between the observed trends and potential associated clinical factors. We also could not associate clusters with risk factors in each geographical area. We have already pointed out the necessity of future studies in this regard.

We were also limited by the level of data granularity. Until 2010, for instance, all registries lacked information on ethnicity and location detail at the level of cantons; limiting the coverage of our spatiotemporal analysis. Similarly, it was not until recently that the issuing of medical certificates of death were made mandatory. Since most Ecuadorians are Catholic, families sometimes take steps to prevent the mention of suicide as the cause of death [9]. Finally, suicide reports lack information about mental health history, as well as familial and/or housing conditions.

The systematic review of Bennett *et al*, recommend coordinated implementation of school-based suicide prevention, prevention of repeat suicide attempts in youth who seek care and who do not seek care; according to sex and gender differences; and ethnic group within a national collaborative youth suicide research-to-practice network [40]. The role of the network would be to identify and facilitate increased implementation of promising programs linked to rigorous evaluation and to eliminate the use of ineffective ones at the regional, provincial, and national level [40]. Wasserman *et al*. concluded that the school environment is the best system we have to perform primary prevention programs designed to improve mental health and give information about unhealthy lifestyles among youth, whilst at the same time raising the general awareness-level about mental health and mental problems [41].

## CONCLUSION

Adolescent suicide rates trend increased during the study period. The spatial analysis indicates the presence of high occurrence clusters in the Amazon and the Southern Highlands regions.

These results alert the Ecuadorian population about the mental health of adolescents and the need to implement programs that counteract major contributors to suicide risk, bullying, and violence in educational centers and familial environments. Our analyses can be used to develop comprehensive strategies to improve the quality of life at a national level by focusing on the most vulnerable: victims of poverty, migration, and ethnic minorities.

## Figures and Tables

**Table 1:** Demographic characteristics and suicide methods in adolescents by sex in Ecuador, 1997-2016.

**Variable**	**Males**		**Females**	
-	10-14 years old n=519 (%)	15-19 years old n=1,480 (%)	10-14 years old n=451 (%)	15-19 years old n=1,374 (%)
**Area**				
**Urban**	356 (68.6)	1030 (69.6)	295 (65.4)	884 (64.3)
**Rural**	150 (28.9)	413 (27.9)	148 (32.8)	433 (31.5)
**Unspecified**	13 (2.5)	37 (2.5)	8 (1.8)	57 (4.1)
**Region**				
**Coast**	129 (24.9)	444 (30.0)	123 (27.3)	338 (24.6)
**Highlands**	361 (69.5)	891 (60.2)	299 (66.3)	900 (65.5)
**Amazon**	29 (5.6)	142 (9.6)	28 (6.2)	135 (9.8)
**Galapágos Islands**	0 (0.0)	3 (0.2)	1 (0.2)	1 (0.1)
**Suicide method**				
**Poisoning:**				
**- Pesticides**	18 (3.5)	192 (13.0)	79 (17.5)	373 (27.1)
**- Other unspecified chemicals and noxious substances**	35 (6.7)	240 (16.2)	114 (25.3)	451 (32.8)
- Others substances*	1(0.2)	23 (1.6)	5 (1.1)	30 (2.2)
**Hanging, strangulation and suffocation**	436 (84.0)	868 (58.4)	228 (50.6)	457 (33.3)
**Drowning and submersion**	5 (1.0)	10 (0.7)	4 (0.9)	1 (0.1)
**Firearm**	20 (3.9)	110 (7.6)	8 (1.8)	36 (2.6)
**Explosive material**	0 (0.0)	1 (0.1)	0 (0.0)	0 (0.0)
**Smoke, fire and flames**	0 (0.0)	0 (0.0)	0 (0.0)	1 (0.1)
**Sharp object**	0(0.0)	3 (0.2)	0 (0.0)	0 (0.0)
**Jumping from a high place**	0(0.0)	7 (0.5)	1 (0.2)	2 (0.1)
**Unspecified means**	4 (0.8)	26 (1.8)	12 (2.7)	23 (1.7)

* Includes groups of ICD 10 codes: X60, X61, X62, X63, X64, X65, X66 and X67

**Table 2:** Joinpoint analysis of suicide rates by sex, age group, and residency area in Ecuador, 1997 to 2016.

**Gender**	**Age group**	**Residency area**	**Rate^a^ 1997**	**Rate^a^ 2016**	**0 Joinpoints**		**1 Joinpoints**		**2 Joinpoint**	
-	-	-	-	-	Year	APC	Year	APC	Year	APC
**Male**	10-14	Urban	2.58	4.54	1997-2016	1.81	1997-2005	13.27*	2004-2016	-4.03*
	10-14	Rural	1.33	4.8	1997-2016	5.60*	1997-2014	4.58	2003-2016	8.51*
	10-14	Total	2.03	3.92	1997-2016	2.11	1997-2007	8.16*	1997-2007	9.91*
	15-19	Urban	9.37	18.72	1997-2016	3.52*	1997-2009	4.84*	1997-2011	5.05*
	15-19	Rural	6.73	14.06	1997-2016	4.83*	1997-2010	4.83*	1997-2010	7.51*
	15-19	Total	8.29	13.76	1997-2016	2.50*	1997-2009	4.03	1997-2010	4.61*
**Female**	10-14	Urban	5.13	4.51	1997-2016	-1.12	2004-2016	-3.85	2004-2016	-5.05*
	10-14	Rural	1.43	5.06	1997-2016	5.95*	2004-2016	7.63*	2002-2016	7.67*
	10-14	Total	3.59	5.85	1997-2016	2.83*				
	15-19	Urban	12.68	9.95	1997-2016	-0.78	2006-2016	-3.73	2004-2016	-3.79*
	15-19	Rural	9.49	9.6	1997-2016	0.31				
	15-19	Total	11.52	12.93	1997-2016	1.44				

APC indicates the estimated annual percent change; *The annual percent change is significantly different from 0 (two-side p < 0.05) ^a^ Suicide rate per 100, 000 population.
